# Effect of Single and Synergistic Reinforcement of PVA Fiber and Nano-SiO_2_ on Workability and Compressive Strength of Geopolymer Composites

**DOI:** 10.3390/polym14183765

**Published:** 2022-09-08

**Authors:** Peng Zhang, Shiyao Wei, Yuanxun Zheng, Fei Wang, Shaowei Hu

**Affiliations:** 1 Yellow River Laboratory, Zhengzhou University, Zhengzhou 450001, China; 2 School of Water Conservancy Engineering, Zhengzhou University, Zhengzhou 450001, China

**Keywords:** workability, compressive strength, geopolymer composites, PVA fiber, Nano-SiO_2_, superplasticizer

## Abstract

Geopolymer composites can be used as a proper substitute for ordinary Portland cement, which can reduce carbon dioxide (CO_2_) emissions and make rational use of industrial waste. In this study, an investigation of the workability and compressive strength of geopolymer composites was carried out through a series of experiments, such as slump flow test, consistency meter test and compressive strength test, to clarify the interaction mechanism among superplasticizer (SP), polyvinyl alcohol (PVA) fiber, Nano-SiO_2_ (NS) and geopolymer composites, thereby improving the properties of engineered composites. The results showed that with the increase in PVA fiber content, the flowability of geopolymer composites decreased, while the thixotropy increased. With the increase in the NS content, the flowability of geopolymer composites first increased and then decreased, reaching its best at 1.0%, while the thixotropy was the opposite. With the increase in the SP content, the flowability of geopolymer composites increased, while the thixotropy decreased. A significant correlation between thixotropy and flowability of geopolymer composites was found (R^2^ > 0.85). In addition, the incorporation of single PVA fiber or NS significantly improved the compressive strength of geopolymer composites. Specifically, the compressive strength of geopolymer composites with 0.8% content PVA fiber (60.3 MPa) was 33.4% higher than that without PVA fiber (45.2 MPa), and the compressive strength of geopolymer composites with 1.5% content NS (52.6 MPa) was 16.4% higher than that without NS (45.2 MPa). Considering the synergistic effect, it is found that the compressive strength of geopolymer composites (58.5–63.3 MPa) was significantly higher than that without PVA fiber (45.2–52.6 MPa). However, the flowability and compressive strength of geopolymer composites were only slightly improved compared to that without NS. With the increase in the SP content, the compressive strength of geopolymer composites showed a trend of a slight decrease on the whole. Consequently, the results of this study may be useful for further research in the field of repair and prevention of the delamination of composite structures.

## 1. Introduction

With the rapid development of the economy, traditional cementitious composites have been widely used in engineering construction, such as tunnel support, highway construction and hydraulic engineering [1]. The production of traditional cement has a great negative impact on the environment. For example, the production process of cement consumes a lot of energy sources and releases CO_2_ [1,2]. Therefore, it is urgently necessary to develop a new type of environmentally friendly cementing material. Some researchers have developed some new materials that could replace traditional cementitious composites, and geopolymer material is the most promising cementing material. In the 1970s, the concept of geopolymer composites was first put forward by Davidovits, whose aim was to investigate a new heat-resistant material in the form of non-flammable plastic materials after various catastrophic fires in France [3]. Geopolymer composite is considered a good substitute for cement because of its low carbon emission, reasonable and effective use of industrial waste, high early strength and acid and alkali resistance [4]. Geopolymer composites are inorganic cementing materials with a three-dimensional network structure, which are mainly composed of alumina tetrahedron (AlO_4_) and silicon–oxygen tetrahedron (SiO_4_). The main raw materials of geopolymer composites are metakaolin (MK), fly ash (FA) and slag [5,6], which are rich in sources, low in price, energy-saving and environmentally friendly in the production process.

However, geopolymer composites and traditional cementitious composites are brittle materials, which are easy to crack and have poor tensile properties [7,8]. By adding fibers, the crack growth of brittle materials can be effectively controlled, and the properties of brittle materials can be improved. At present, the most commonly used fibers include steel fibers, carbon fibers, polypropylene fibers and PVA fibers [9,10]. The strengthening effect of steel fiber on the matrix is very obvious, but the steel fiber is easy to be rusted and its price is relatively high [11,12,13]. Carbon fiber has the advantages of low specific gravity and large elastic modulus, high temperature resistance, and not being easy to corrode, but it also has the disadvantages of poor toughness and insufficient impact resistance [14,15]. Polypropylene fiber has the characteristics of strong toughness, chemical stability, excellent acid and alkali resistance and being easy to disperse uniformly in the matrix, but its elastic modulus and tensile strength are low [16]. However, as a kind of polymer synthetic material, PVA fiber has high elastic modulus and high tensile strength, and its surface hydrophilic property determines the high adhesion between fiber and matrix, which is beneficial to the improvement in toughness of composites [17,18,19,20]. Therefore, it is feasible to use PVA fiber for improving the performance of geopolymer composites.

In recent years, some scholars have begun to carry out research on PVA fiber reinforced geopolymer composites. Wang [21] investigated the strain hardening geopolymer composites (SHGC) reinforced by PVA-steel hybrid fibers. The findings suggested that the incorporation of PVA and recycled steel hybrid fibers led to an obvious decrease in the fluidity, solidification time and bending strength of SHGC, while the shrinkage resistance and compressive strength were greatly increased. Trindade et al. [22] demonstrated that the incorporation of PVA fiber could improve the compression performance, ductility and tensile strength of potassium-based and sodium-based SHGC, and play the role of internal micro-restraint. Zahid et al. [23] found that PVA fiber had an obvious effect on the fracture behavior of geopolymer composites. After cracking, the behavior of geopolymer composites with 0.04 mm diameter PVA fiber was better than that of geopolymer composites with 0.2 mm diameter PVA fiber and met the pseudo-strain hardening standard. Cheng et al. [19] researched the influences of carbon nanotubes and steel-PVA hybrid fibers on lightweight geopolymer composites and found that the incorporation of carbon nanotubes and hybrid fibers would reduce the fluidity of lightweight geopolymer composites, while the incorporation of PVA fiber, which plays a role in bridging cracks, obviously enhanced the tensile properties of lightweight geopolymer composites. Some scholars have pointed out that the addition of Nano-materials would strengthen the performance of geopolymer composites to some extent. Nano-materials mainly include NS, carbon nanotubes and Nano-CaCO_3_ [24]. Among them, NS has good pozzolanic activity, nucleation effect and filling effect [25,26]; thus, using NS to improve the properties of geopolymer composites has gradually become the focus among some scholars. Luo et al. [27] found that the addition of NS could strengthen the macro-performance of geopolymer composites better, while Nano-titanium improved the micro-mechanical properties better. Moreover, the use of NS and Nano-titanium would increase the early reaction rate of geopolymers. Zhang et al. [28] studied the mechanical properties and resistance to chloride ion penetration of PVA fiber and NS reinforced geopolymer composites.

The workability and thixotropy of fresh concrete are important properties of concrete that are relevant to a series of practical applications of concrete, such as mixing, transportation and pumping [29]. They are also related to the mechanical performance and durability of hardened concrete. Archez et al. [30] studied the effects of wollastonite and glass fiber on the workability of geopolymer mortars. The results showed that wollastonite could increase the viscosity of geopolymer composites, while glass fiber would cause a decrease in the ductility of geopolymer composites. In addition, the viscosity and solidification time of the fresh mixture could be determined by the aluminum content. Xu et al. [31] demonstrated that high calcium basalt fiber could increase the initial setting time and final setting time and decrease the fluidity of slag and FA-based geopolymer composite. Junior [32] found that the workability of geopolymer composites mixed with polypropylene fiber was better than that with glass fiber or PVA fiber because it was dispersed better in geopolymer composites. Panda et al. [33] demonstrated that the addition of nano-clay would enhance the thixotropy of the geopolymer mixture, while the thixotropy effect of nano-clay could be eliminated when the content of the alkali activator was relatively high. Bong et al. [34] studied the properties of geopolymer with wollastonite microfibers. In the experiment, the replacement rates of wollastonite with sand were 0%, 5%, 10%, 15%, 20% and 30%. The results showed that the thixotropy and static yield stress of the mixture were enhanced at 10% replacement. In addition, as a structural material, concrete must have sufficient strength to resist the destruction of various loads. Moreover, the compressive strength of concrete as a brittle material is much higher than other strengths [35]. Therefore, to ensure the quality of concrete engineering, and improve engineering efficiency, it is of great significance to control the compressive strength of concrete [36,37,38]. Gholampour et al. [39] found that the incorporation of blast furnace slag would improve the compressive strength and tensile strength of geopolymer mortar because it could reduce the pores in the transition zone between cementing materials and sand. Yeddula et al. [40] demonstrated that the compressive strength of ferric silicate-based geopolymer composites was 112.4% higher than that of silicate mortar. Moreover, the geopolymer mortar cured in the oven was easier for obtaining better strength growth than that cured at room temperature. However, existing studies have rarely investigated the relationship between the workability and thixotropy of geopolymer composites. The workability of geopolymer composites can be tested conveniently and accurately, but the testing of thixotropy parameters of geopolymer composites requires relatively good equipment, which is relatively expensive and not suitable for the construction site. The establishment of the relationship between flowability and thixotropy would provide a good way to solve this problem.

Although there are many investigations into the effects of nanoparticles and fibers on the performance of geopolymer composites, there are few studies on the single and synergistic reinforcement of PVA fiber and NS on geopolymer composites. Therefore, in this study, the workability and compressive strength of geopolymer composites with SP and the single and synergistic reinforcement of PVA fiber and NS were mainly investigated through a series of experiments such as slump flow test, consistency meter test and compressive strength test to clarify the interaction mechanism among SP, PVA fiber, NS and geopolymer composites, thereby improving the properties of engineered composites.

## 2. Experiments

### 2.1. Materials

The properties of geopolymer composite materials include MK, FA, NS and PVA fibers and are presented in Table 1. The PVA fiber and NS used in the experiment were produced by Kuraray Company of Japan and Hangzhou Wanjing New Material Company of China, respectively. The alkali activator solution was composed of sodium hydroxide (NaOH) (99% purity), waterglass (34.3% solid content and 3.2 modulus) and water. According to Sun [41], the waterglass modulus was tuned up to 1.3 through incorporating NaOH; afterwards, the mass fraction of sodium oxide was adjusted to 15% by adding water. Moreover, the SP with a water-reducing rate of 21% and the 75–120 μm quartz sand were also used in this study.

### 2.2. Mix Proportions

During material mixing, the water-binder ratio was set as 0.65, the binder ratio was set as 1.0 and the mass ratio of FA to MK was 3 to 7 according to Zhang [25]. In the test, the effects of PVA fiber, NS and SP were considered. Therefore, the content of PVA was set to 0%, 0.2%, 0.4%, 0.6%, 0.8%, 1.0% and 1.2% (volume fraction), the content of NS was set to 0%, 0.5%, 1.0%, 1.5%, 2.0% and 2.5% (mass fraction of cementing materials), and the content of SP was set to 0%, 0.25%, 0.5%, 0.75% and 1.0% [42]. In this study, a total of 26 mixing ratios were set, as listed in Table 2.

### 2.3. Mixture Preparation

In the preparation of PVA fibers and NS reinforced geopolymer composite, PVA fiber and NS should be uniformly dispersed in the matrix to fully enhance the properties of the geopolymer composites. To meet the test requirements, it is necessary to choose appropriate preparation methods and processes. First, MK, FA and quartz sand were dry-mixed for 2 min, then the PVA fibers were added in two batches and mixed for 2 min each time to make the PVA fibers disperse evenly. Subsequently, an alkali activator was added and stirred for 2 min. Finally, NS, water and SP were blended and then stirred for 2 min to ensure the uniform dispersion of NS powder. After the preparation of the geopolymer composites, the mixture was loaded into a slump tube and consistency meter, respectively, to test the working performance and thixotropy. Meanwhile, the rest of the mixture was loaded into the test mold, and 24 h later, the specimens were taken out of the molds and placed into a standard curing box for 28 d. Finally, a compressive strength test was carried out on the specimens.

### 2.4. Slump Flow Test

A slump flow test was carried out to measure the flowability of geopolymer composites according to GB/T 50080-2016 standard procedure [43]. A slump tube with a top diameter of 100 mm, a height of 300 mm and a bottom diameter of 200 mm was used in the test. The fresh geopolymer composites were loaded into the slump tube, and air was removed from the composite by a rod. After that, the surface of the geopolymer composites was flattened and the slump tube was vertically lifted within 5–10 s to allow the geopolymer composites to flow out of the tube. The ultimate results were mean values of two tests for each mixture.

### 2.5. Consistency Meter Test

**Figure 1:**
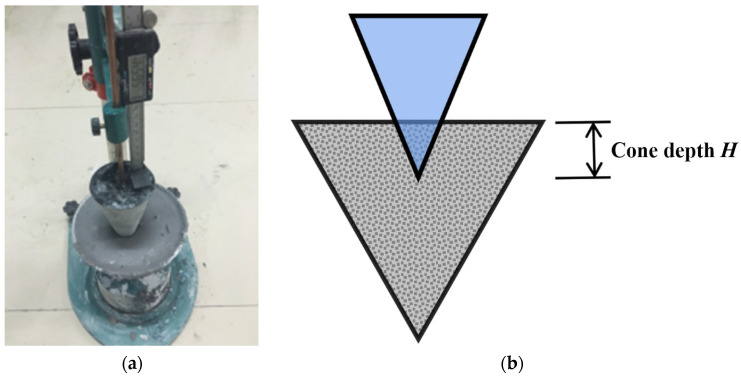
Consistency meter (**a**) Device image; (**b**) Schematic diagram.

A consistency meter (Figure 1) was used to test the thixotropy of geopolymer composites [44]. The procedure was as follows:

(i) The fresh geopolymer composites were put into the consistency meter container, then a rod was used to remove the air and the surface was flattened; the cone and ruler were adjusted to make the cone tip come into contact with the geopolymer composites’ surface.
(ii) The bolt fixing the cone was loosened to make the cone freely insert into the geopolymer composites, then the bolt was tightened and the cone depth $H_{1}$ was measured.
(iii) The cone and ruler were reset, a rod was used to remove the air and the surface was flattened.
(iv) After 10 min, the cone depth $H_{2}$ was measured according to the above steps. The cone depth $H_{3}$ was obtained in the same way.
(v) The difference in cone depth $\Delta H=H_{3}-H_{2}$ was calculated, and the thixotropy of fresh geopolymer composites could be characterized by ΔH.

### 2.6. Compressive Strength Test

A compressive strength test of PVA fiber and NS reinforced geopolymer composites was carried out according to JGJ/T 70-2009 standard procedure [45]. Three cube specimens with a side length of 70.7 mm were prepared for each mixture. The test was conducted on a 2000 kN universal testing machine produced by Shanghai Hualong Company of China, and the loading rate was always 1.5 kN/s. The average of the three specimens was taken as the final compressive strength.

## 3. Results and Discussion

### 3.1. Flowability

**Figure 2:**
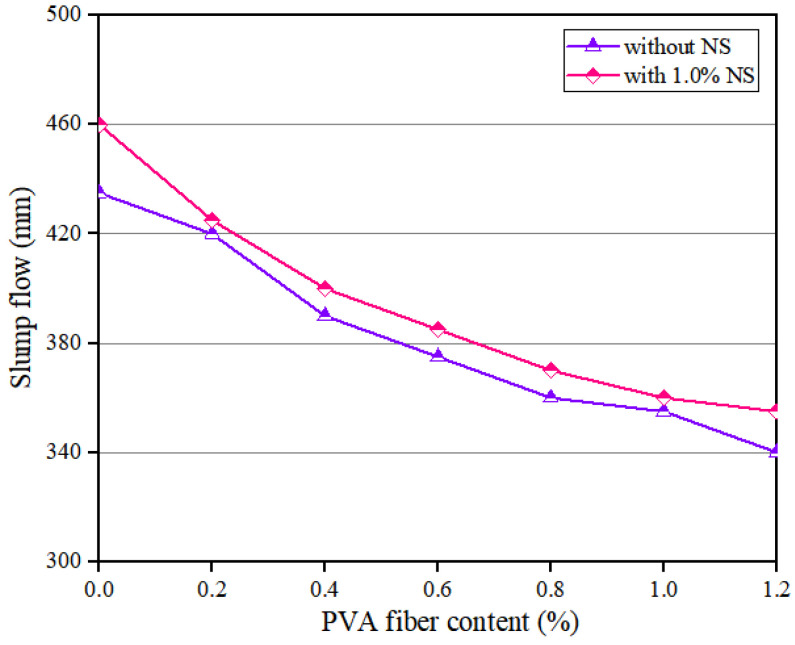
Influence of PVA fiber on flowability of geopolymer composites.

The flowability of geopolymer composites with different PVA fiber contents is shown in Figure 2. The results showed that the slump flow of the geopolymer composites continuously decreased with the increase in PVA fiber content, which is consistent with the results obtained by Shah et al. [46]. Specifically, when the content of NS was 0, with the growth of PVA fiber content, the slump flow of the geopolymer composites decreased by 21.84%, from 435 mm to 340 mm. When the content of NS was 1.0%, as the content of PVA fiber increased, the slump flow of the geopolymer composites decreased by 22.83%, from 460 mm to 355 mm. The above experimental results are related to the following three reasons. Firstly, the hydrophilicity of hydroxyl groups in the molecules of PVA fiber made this fiber absorb a large amount of free water in the geopolymer composites, thus reducing the fluidity of the geopolymer composites. Secondly, the incorporation of PVA fiber increased the internal pores of the geopolymer composites, which reduced the uniformity of these composites, thus reducing the fluidity of geopolymer composites. Thirdly, an increase in the PVA fiber content will require more slurry to wrap the fibers, further reducing the fluidity of the geopolymer composite [47,48,49,50]. In addition, when the PVA fiber dosage was the same, the slump flow of geopolymer composites with 1.0% content NS was higher than that of geopolymer composites with 0 content NS.

**Figure 3:**
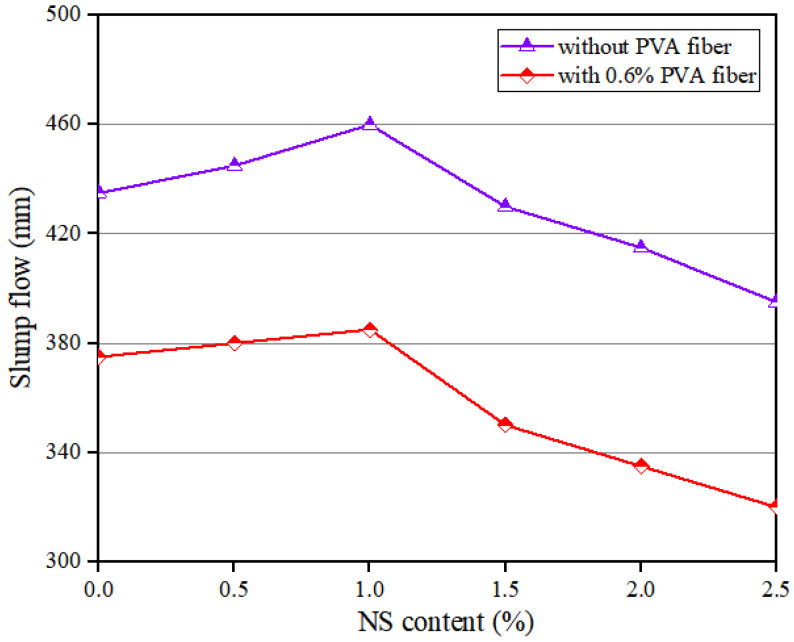
Influence of NS on flowability of geopolymer composites.

Figure 3 shows that when the NS dosage was enhanced, the slump flow of the geopolymer composites rose continuously before 1.0% NS; afterwards, it began to decline. Specifically, when the content of PVA fiber was 0%, with the increase in NS content, the slump flow increased by 5.75%, from 435 mm to 460 mm, and then decreased by 14.13%, from 460 mm to 395 mm. When the content of PVA fiber was 0.6%, with the increase in NS content, the slump flow increased by 2.67%, from 375 mm to 385 mm, and then decreased by 16.88%, from 385 mm to 320 mm. The above results are consistent with those of Zhang et al. [51]. When the content of NS was less than 1.0%, the reason why the fluidity of the composites increased when the NS dosage increased is that NS could fill the voids in mixtures, making the geopolymer composites more lubricated [42]. In addition, NS would react in the mixture to reduce the water absorption of NS. When the content of NS was more than 1.0%, the fluidity of the geopolymer composites decreased with the increase in NS content. The reason was that the specific surface area of NS was large, which caused the increase in the amount of water adsorbed by the surface of the mixture [52].

**Figure 4:**
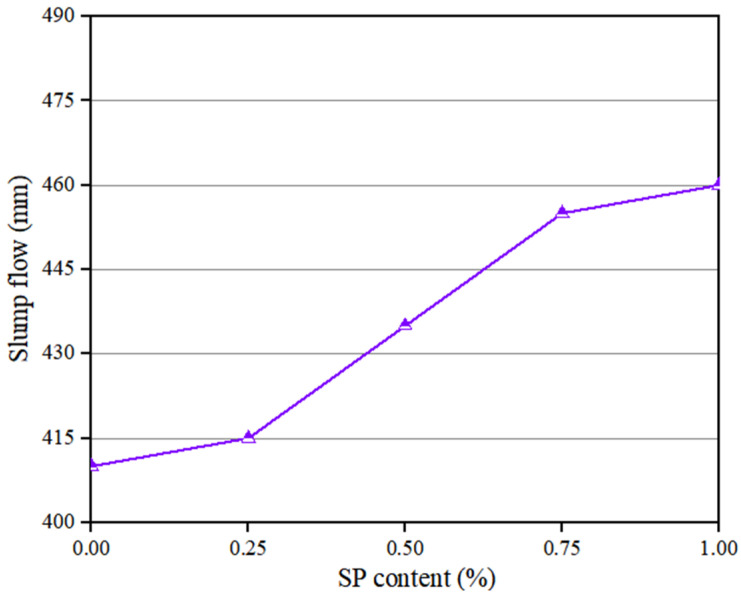
Influence of SP on flowability of geopolymer composites.

The flowability of PVA fibers and NS reinforced geopolymer composite with different SP contents is shown in Figure 4. It can be seen that the slump flow of the geopolymer composites rose with the increase in SP dosage. This is similar to the findings of Memon et al. [53]. With the increase in the SP content from 0% to 1.0%, the slump flow of the geopolymer composites increased by 12.2%, from 410 mm to 460 mm. These changes in the flowability parameters were because all kinds of particles in the geopolymer composites had the same symbolic electric charge. Under the combined action of electrical repulsion and the lubrication of SP, the system was in a relatively stable suspension state, and the flocculating structure was then disintegrated to release the free water [54,55,56].

### 3.2. Thixotropy

**Figure 5:**
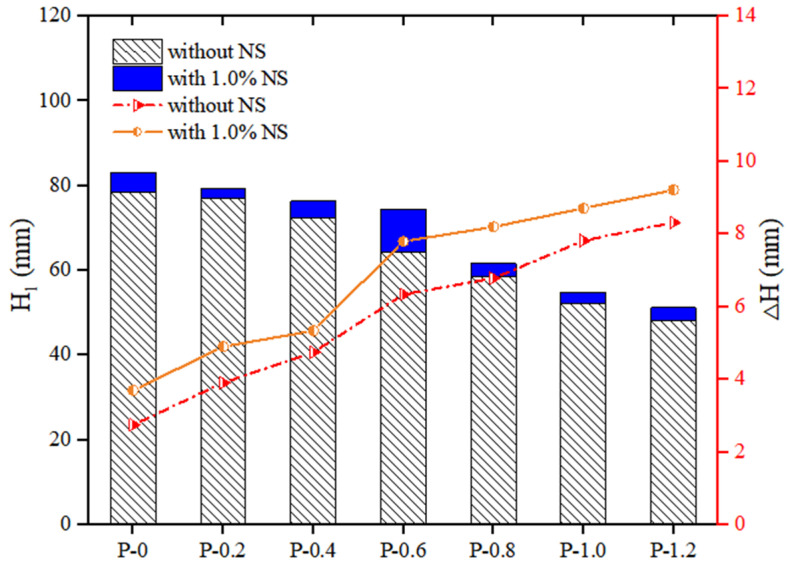
Influence of PVA fibers on thixotropy of geopolymer composites.

The thixotropy of PVA fibers and NS strengthened geopolymer composites with different fiber contents is shown in Figure 5. It could be seen that with the increase in the content of PVA fiber, the cone depth $H_{1}$ of the geopolymer composites decreased, while the difference in cone depth ΔH increased. When the content of NS was 0, as the content of PVA fiber increased from 0% to 1.2%, the $H_{1}$ of the geopolymer composites decreased by 38.37%, from 78.36 mm to 48.29 mm, and the ΔH of the geopolymer composites increased by 149.19%, from 3.70 mm to 9.22 mm. When the content of NS was fixed at 1.0%, as the content of PVA fiber increased from 0% to 1.2%, the $H_{1}$ of the geopolymer composites decreased by 38.39%, from 83.11 mm to 51.20 mm, and the ΔH of the geopolymer composites increased by 201.82%, from 2.75 mm to 8.30 mm. These changes in the thixotropy parameters were because the incorporation of PVA fiber accelerated the formation of the flocculation structure in the geopolymer composites. Therefore, the thixotropy of geopolymer composites increased with the increase in the content of PVA fiber [57].

**Figure 6:**
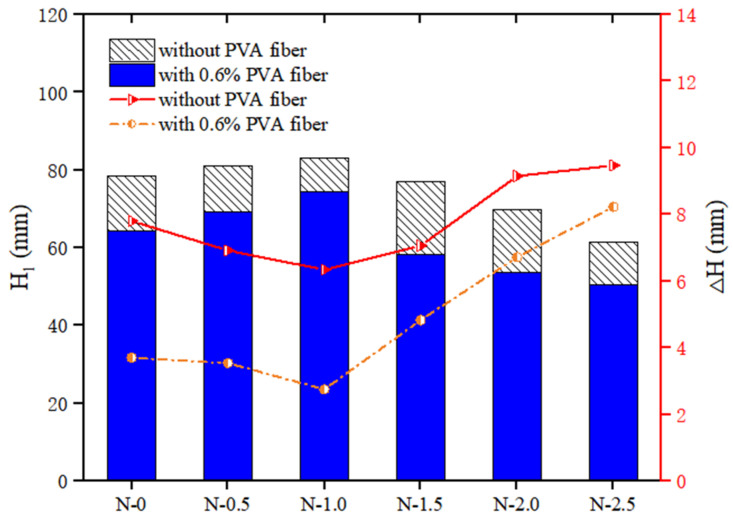
Effect of NS on thixotropy of geopolymer composites.

Figure 6 shows that when the volume of PVA fiber content was fixed at 0, as the NS dosage rose, the cone depth $H_{1}$ of the geopolymer composites first increased from 78.36 mm to 83.11 mm, and then decreased to 61.42 mm. However, ΔH first decreased from 3.70 mm to 2.75 mm, and then increased to 8.23 mm. When the PVA fiber content was fixed at 0.6%, as the NS content increased, the cone depth $H_{1}$ first increased from 78.36 mm to 74.40 mm, and then decreased to 50.45 mm, while ΔH first decreased from 7.80 mm to 6.35 mm, and then increased to 9.47 mm.

**Figure 7:**
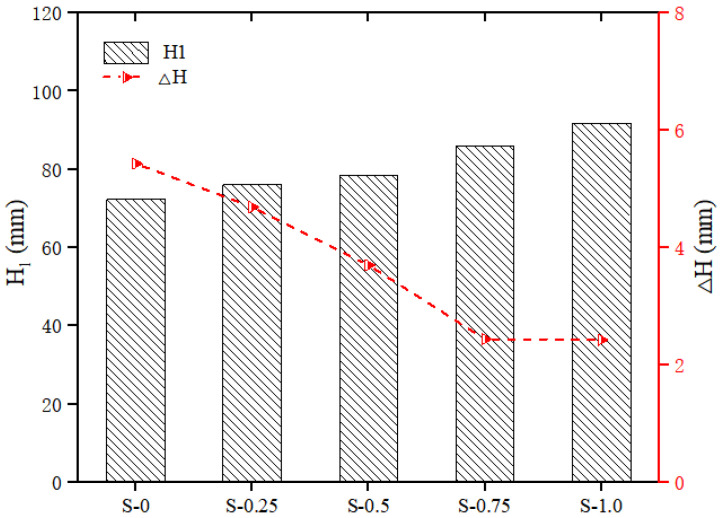
Effect of SP on thixotropy of geopolymer composites.

The thixotropy of the geopolymer composites with different SP contents is shown in Figure 7. With the increase in the SP content, the cone depth $H_{1}$ of the geopolymer composites increased, while the cone depth difference ΔH decreased. Specifically, as the content of SP increased from 0 to 1.0%, the $H_{1}$ of the geopolymer composites increased by 27.02%, from 72.32 mm to 91.86 mm, while the ΔH of the geopolymer composites decreased by 55.25%, from 5.43 mm to 2.43 mm. These changes in the thixotropy parameters were because, with the increase in the SP content, the slurry changed from the original flocculation structure to the coexistence of dispersed particles and a non-dispersible flocculation structure. Therefore, the thixotropy of geopolymer composites was reduced [42].

### 3.3. Relationship between Thixotropy and Flowability of Geopolymer Composites

**Figure 8:**
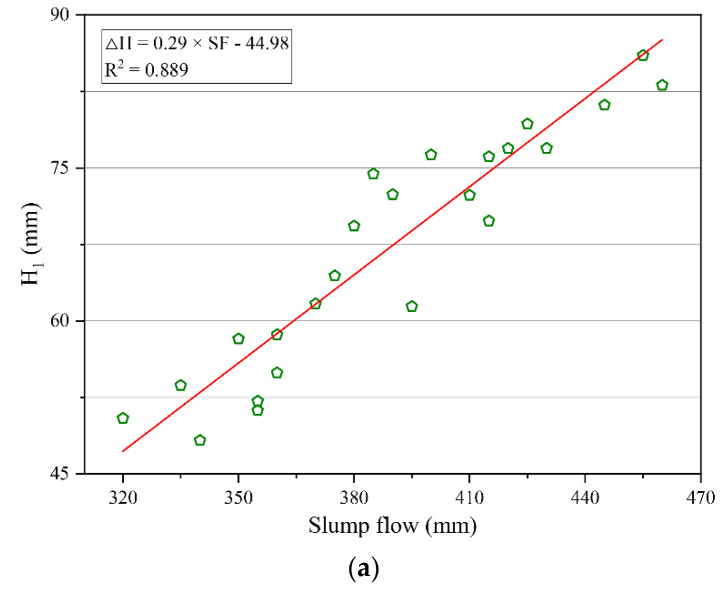

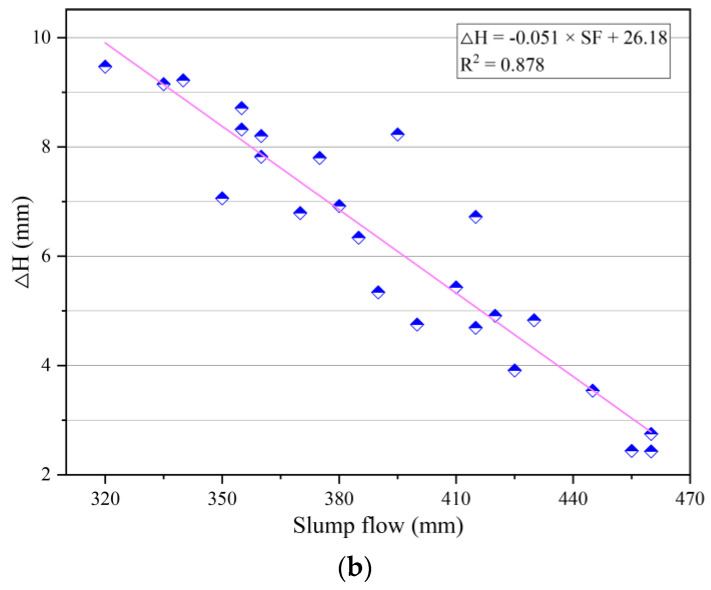
Correlation between thixotropy and flowability parameters of geopolymer composites. (**a**) Correlation between the cone depth *H_1_* and slump flow. (**b**) Correlation between the cone depth difference Δ*H* and slump flow.

The fitting analysis of thixotropy and flowability of geopolymer composites was carried out according to the cone depth $H_{1}$ and the cone depth difference ΔH obtained by the consistency meter test and slump flow obtained by the slump flow test, as shown in Figure 8. It can be seen from Figure 8 that there was a significant linear correlation between the thixotropy parameters and flowability parameters of the geopolymer composites. Specifically, the cone depth $H_{1}$ had a positive correlation with the slump flow, while the cone depth difference ΔH had a negative correlation with the slump flow. In addition, the correlation coefficient R^2^ in the fitting results was less than 0.9, which indicates that there were certain deviations in the correlation between the parameters of thixotropy and slump flow. The reason for this phenomenon may be that the testing time of the consistency meter test was long, causing a decrease in temperature during the determination, which would change the thixotropy of the geopolymer composites.

### 3.4. Compressive Strength

**Figure 9:**
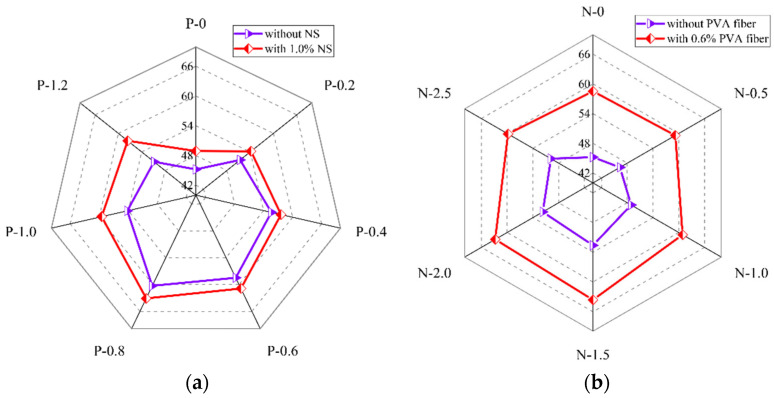

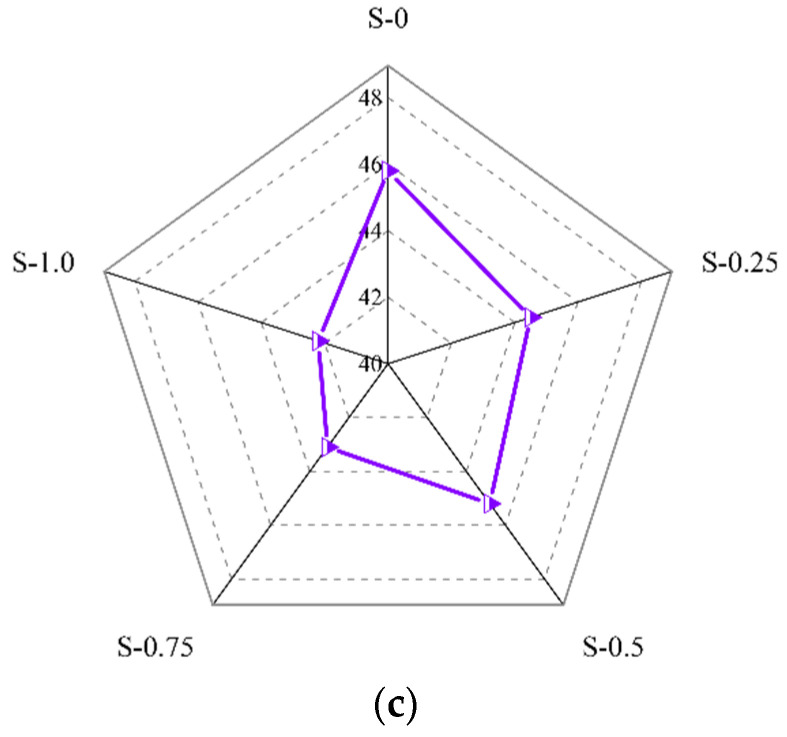
Influence of (**a**) PVA fiber, (**b**) NS, and (**c**) SP on compressive strength of geopolymer composites.

The compressive strength of PVA fiber and NS reinforced geopolymer composites with different PVA fiber contents is shown in Figure 9a. It could be seen that when the NS dosage was fixed, with the growth in PVA fiber dosage, the compressive strength of the geopolymer composites first increased and then decreased, reaching the biggest at 0.8% PVA fiber. The results presented in Figure 9a are similar to those of Zerfu and Ekaputri [58]. When the NS dosage was 0, as the PVA fiber dosage increased, the compressive strength first increased from 45.2 MPa to 60.3 Mpa, and then decreased to 50.8 Mpa. When the content of NS was 1.0%, with the increase in the content of PVA fiber, the compressive strength first increased from 48.9 Mpa to 63.1 Mpa, and then decreased to 57.6 Mpa. Overall, the PVA fibers continuously increased the compressive strength of the geopolymer composites before 0.8% dosage. It was due to this that PVA fibers could prevent the generation and expansion of microcracks caused by condensation hardening shrinkage in the matrix. Meanwhile, the increase in the content of PVA fiber will strengthen the three-dimensional support system formed between the fiber and the matrix, thereby improving the compressive strength of the geopolymer composites [47,59,60]. When the fiber content exceeded 0.8%, the excess fibers were not easily uniformly dispersed due to agglomeration, which leads to the existence of a large number of pores in the matrix, thereby reducing the strengths of the geopolymers [61].

The compressive strength of PVA fiber and NS reinforced geopolymer composites with different NS contents is shown in Figure 9b. It could be seen that when the PVA fiber content was fixed, with the increase in NS content, the compressive strength of the geopolymer composites first increased and then decreased, reaching the biggest at 1.5% NS content. When the content of PVA fiber was 0, with the increase in NS content, the compressive strength of the geopolymer composites increased from 45.2 MPa to 52.6 MPa, and then decreased to 49.8 MPa. When the content of PVA fiber was 0.6%, with the increase in the content of NS, the compressive strength of the geopolymer composites increased from 58.5 MPa to 63.6 MPa, and then decreased to 59.8 MPa. The compressive strength of the geopolymer composites was positively correlated with the content of NS before 1.5% NS. The reason for the above phenomenon is that the fine-grained NS could fill the voids in the matrix of geopolymer composites and make the matrix denser [52,62]. When the content of NS was more than 1.5%, the content of NS had a negative effect on the compressive strength of the geopolymer composites. The reason for this phenomenon is that the specific surface area of NS particles was large, therefore needing lots of water for the reaction. When the NS content exceeds the optimal value, the geopolymer composites are difficult to stir evenly, resulting in an insufficient internal reaction of the composites and a loose structure of the geopolymer composites.

Figure 9c shows that, as the SP contents increased, the compressive strength of the geopolymer composites showed a slight downward trend. Specifically, when the content of SP increased from 0% to 1.0%, the compressive strength of the geopolymer composites decreased by 7.64%, from 45.8 MPa to 42.2 MPa. This was because the water-binder ratio remained unchanged in this experiment, while the incorporation of SP could lead to the precipitation of water in the geopolymer composites.

## 4. Conclusions

In this study, the effects of SP and the single and synergistic reinforcement of PVA fibers and NS on the workability and compressive performance of geopolymer composites were investigated. The main conclusions are as follows:

(i) The incorporation of PVA fiber decreased the flowability of geopolymer composites, while the incorporation of SP increased the flowability of geopolymer composites. When the NS content increased, the flowability of geopolymer composites first increased and then decreased, reaching its best at 1.0%. In addition, the effect of the incorporation of PVA, NS and SP on the thixotropy of geopolymer composites was opposite to that on the flowability.
(ii) There was a significant linear correlation between thixotropy and flowability of geopolymer composites (R^2^ > 0.85). Specifically, the cone depth $H_{1}$ had a positive correlation with the slump flow, while the cone depth difference ΔH had a negative correlation with the slump flow.
(iii) The incorporation of single PVA fiber or NS would increase the compressive strength of geopolymer composites. Specifically, the compressive strength of geopolymer composites with 0.8% PVA fiber was 33.4% higher than that without PVA fiber, and the compressive strength of geopolymer composites with 1.5% NS was 16.4% higher than that without NS. Moreover, when the PVA fibers and NS coexist in geopolymer composites, 0.6% PVA significantly improved the compressive strength of geopolymer composites, while 1.0% NS could slightly improve the compressive strength of geopolymer composites.

In this study, the effect of a single and synergistic reinforcement of PVA fiber and Nano-SiO_2_ on the workability and compressive strength of geopolymer composites was investigated, which has great significance in the application of geopolymer composites. However, the exploration of the relationship between flowability and thixotropy is essential for the restoration of buildings, and the properties of geopolymer composites are affected by the chemical composition of binders, curing conditions and environmental conditions; thus, these related works can be carried out in future research.

## Figures and Tables

**Table 1:** Properties of NS and PVA fiber.

	Specific Surface Area (m^2^/g)	Stacking Density (g/cm^3^)	pH	Nominal Particle Size (nm)	Loss on Ignition (%)
NS	200	0.054	6.21	30	1.0
	**Fiber** **Length (mm)**	**Filament** **Diameter (μm)**	**Elongation at Fracture (%)**	**Flexural Strength (MPa)**	
PVA fiber	12	40	6.5	1560	

**Table 2:** Mix proportions of geopolymer composites.

Mix No.	Waterglass	NaOH	Water	Sand	MK	FA	PVA Fiber %	NS %	SP
	kg/m^3^								kg/m^3^
P-0.2	445.4	71	106.2	613.6	429.5	184.1	0.2	0	3.07
P-0.4	445.4	71	106.2	613.6	429.5	184.1	0.4	0	3.07
P-0.6	445.4	71	106.2	613.6	429.5	184.1	0.6	0	3.07
P-0.8	445.4	71	106.2	613.6	429.5	184.1	0.8	0	3.07
P-1.0	445.4	71	106.2	613.6	429.5	184.1	1.0	0	3.07
P-1.2	445.4	71	106.2	613.6	427.2	183.1	1.2	0	3.07
N-0.5	445.4	71	106.2	613.6	425.0	182.2	0	0.5	3.07
N-1.0	445.4	71	106.2	613.6	422.7	181.2	0	1.0	3.07
N-1.5	445.4	71	106.2	613.6	420.4	180.2	0	1.5	3.07
N-2.0	445.4	71	106.2	613.6	425.0	182.2	0	2.0	3.07
N-2.5	445.4	71	106.2	613.6	425.0	182.2	0	2.5	3.07
PN-0.2-1.0	445.4	71	106.2	613.6	425.0	182.2	0.2	1.0	3.07
PN-0.4-1.0	445.4	71	106.2	613.6	425.0	182.2	0.4	1.0	3.07
PN-0.6-1.0	445.4	71	106.2	613.6	425.0	182.2	0.6	1.0	3.07
PN-0.8-1.0	445.4	71	106.2	613.6	427.2	183.1	0.8	1.0	3.07
PN-1.0-1.0	445.4	71	106.2	613.6	422.7	181.2	1.0	1.0	3.07
PN-1.2-1.0	445.4	71	106.2	613.6	420.4	180.2	1.2	1.0	3.07
PN-0.6-0.5	445.4	71	106.2	613.6	425.0	182.2	0.6	0.5	3.07
PN-0.6-1.5	445.4	71	106.2	613.6	420.4	180.2	0.6	1.5	3.07
PN-0.6-2.0	445.4	71	106.2	613.6	425.0	182.2	0.6	2.0	3.07
PN-0.6-2.5	445.4	71	106.2	613.6	420.4	180.2	0.6	2.5	3.07
S-0	445.4	71	106.2	613.6	429.5	184.1	0	0	1.84
S-0.25	445.4	71	106.2	613.6	429.5	184.1	0	0	2.46
S-0.5	445.4	71	106.2	613.6	429.5	184.1	0	0	3.07
S-0.75	445.4	71	106.2	613.6	429.5	184.1	0	0	3.68
S-1	445.4	71	106.2	613.6	429.5	184.1	0	0	4.30

## References

[1] Yang H.M., Zhang S.M., Lei W., Chen P., Shao D.K., Tang S.W., Li J.Z. (2022). High-ferrite Portland cement with slag: Hydration, microstructure, and resistance to sulfate attack at elevated temperature. Cement Concrete Comp..

[2] Peng Y., Tang S., Huang J., Tang C., Liu Y. (2022). Fractal analysis on pore structure and modeling of hydration of magnesium phosphate cement paste. Fractal Fract..

[3] Davidovits J. (1994). Properties of geopolymer cements. First International Conference on Alkaline Cements and Concretes.

[4] Oderji S.Y., Chen B., Ahmad M.R., Shah S. (2019). Fresh and hardened properties of one-part fly ash-based geopolymer binders cured at room temperature: Effect of slag and alkali activators. J. Clean. Prod..

[5] Xiao R., Ma Y.T., Jiang X., Zhang M.M., Zhang Y.Y., Wang Y.H., Huang B.S., He Q. (2020). Strength, microstructure, efflorescence behavior and environmental impacts of waste glass geopolymers cured at ambient temperature. J. Clean. Prod..

[6] Bajpai R., Choudhary K., Srivastava A., Sangwan K.S., Singh M. (2020). Environmental impact assessment of fly ash and silica fume based geopolymer concrete. J. Clean. Prod..

[7] Wang L., Li G.X., Li X., Guo F.X., Lu X., Hanif A. (2022). Influence of reactivity and dosage of MgO expansive agent on shrinkage and crack resistance of face slab concrete. Cement Concrete Comp..

[8] Wang L., Yu Z., Liu B., Zhao F., Jin M. (2022). Effects of fly ash dosage on shrinkage, crack resistance and fractal characteristics of face slab concrete. Fractal Fract..

[9] Ranjbar N., Zhang M.Z. (2020). Fiber-reinforced geopolymer composites: A review. Cement Concrete Comp..

[10] Zheng Y.X., Zhuo J.B., Zhang P., Ma M. (2022). Mechanical properties and meso-microscopic mechanism of basalt fiber-reinforced recycled aggregate concrete. J. Clean. Prod..

[11] Liu Y.W., Zhang Z.H., Shi C.J., Zhu D.J., Li N., Deng Y.L. (2020). Development of ultra-high performance geopolymer concrete (UHPGC): Influence of steel fiber on mechanical properties. Cement Concrete Comp..

[12] Guo X.L., Xiong G.Y. (2021). Resistance of fiber-reinforced fly ash-steel slag based geopolymer mortar to sulfate attack and drying-wetting cycles. Constr. Build. Mater..

[13] Wen C.C., Zhang P., Wang J., Hu S.W. (2022). Influence of fibers on the mechanical properties and durability of ultra-high-performance concrete: A review. J. Build. Eng..

[14] Chu S.H., Ye H., Huang L., Li L.G. (2021). Carbon fiber reinforced geopolymer (FRG) mix design based on liquid film thickness. Constr. Build. Mater..

[15] Ma S.Q., Yang H.L., Zhao S.J., He P.G., Zhang Z.H., Duan X.M., Yang Z.H., Jia D.C., Zhou Y. (2021). 3D-printing of architectured short carbon fiber-geopolymer composite. Compos. Part B-Eng..

[16] Humur G., Cevik A. (2022). Effects of hybrid fibers and nanosilica on mechanical and durability properties of lightweight engineered geopolymer composites subjected to cyclic loading and heating-cooling cycles. Constr. Build. Mater..

[17] Kan L.L., Wang W.S., Liu W.D., Wu M. (2020). Development and characterization of fly ash based pva fiber reinforced engineered geopolymer composites incorporating metakaolin. Cement Concrete Comp..

[18] Trindade A., Heravi A.A., Curosu I., Liebscher M., Silva F.D., Mechtcherine V. (2020). Tensile behavior of strain-hardening geopolymer composites (SHGC) under impact loading. Cement Concrete Comp..

[19] Cheng Z.J., Liu Z.Z., Hao H.L., Lu Y.Y., Li S. (2022). Multi-scale effects of tensile properties of lightweight engineered geopolymer composites reinforced with MWCNTs and steel-PVA hybrid fibers. Constr. Build. Mater..

[20] Zhang P., Wei S., Wu J., Zhang Y., Zheng Y. (2022). Investigation of mechanical properties of PVA fiber-reinforced cementitious composites under the coupling effect of wet-thermal and chloride salt environment. Case. Stud. Constr. Mat..

[21] Wang Y., Chan C.L., Leong S.H., Zhang M.Z. (2020). Engineering properties of strain hardening geopolymer composites with hybrid polyvinyl alcohol and recycled steel fibres. Constr. Build. Mater..

[22] Trindade A., Curosu I., Liebscher M., Mechtcherine V., Silva F.D. (2020). On the mechanical performance of K- and Na-based strain-hardening geopolymer composites (SHGC) reinforced with PVA fibers. Constr. Build. Mater..

[23] Zahid M., Shafiq N., Razak S., Tufail R.F. (2020). Investigating the effects of NaOH molarity and the geometry of PVA fibers on the post-cracking and the fracture behavior of engineered geopolymer composite. Constr. Build. Mater..

[24] Golewski G.L., Szostak B. (2021). Strengthening the very early-age structure of cementitious composites with coal fly ash via incorporating a novel nanoadmixture based on C-S-H phase activators. Constr. Build. Mater..

[25] Gao Z., Zhang P., Wang J., Wang K.X., Zhang T.H. (2022). Interfacial properties of geopolymer mortar and concrete substrate: Effect of polyvinyl alcohol fiber and Nano-SiO_2_ contents. Constr. Build. Mater..

[26] Zhang P., Wang W.S., Lv Y.J., Gao Z., Dai S.Y. (2022). Effect of polymer coatings on the permeability and chloride ion penetration resistances of nano-particles and fibers-modified cementitious composites. Polymers.

[27] Luo Z.Y., Li W.G., Gan Y.X., He X.Z., Castel A., Sheng D.C. (2021). Nanoindentation on micromechanical properties and microstructure of geopolymer with Nano-SiO_2_ and Nano-TiO_2_. Cement Concrete Comp..

[28] Zhang X.M., Zhang P., Wang T.Y., Zheng Y., Qiu L.H., Sun S.W. (2022). Compressive strength and anti-chloride ion penetration assessment of geopolymer mortar merging PVA fiber and Nano-SiO_2_ using RBF–BP composite neural network. Nanotechnol. Rev..

[29] Szostak B., Golewski G.L. (2021). Rheology of cement pastes with siliceous fly ash and the CSH nano-admixture. Materials.

[30] Archez J., Texier-Mandoki N., Bourbon X., Caron J.F., Rossignol S. (2020). Rossignol, Influence of the wollastonite and glass fibers on geopolymer composites workability and mechanical properties. Constr. Build. Mater..

[31] Xu J., Kang A.H., Wu Z.G., Xiao P., Gong Y.F. (2021). Effect of high-calcium basalt fiber on the workability, mechanical properties and microstructure of slag-fly ash geopolymer grouting material. Constr. Build. Mater..

[32] Junior J., Saha A.K., Sarker P.K., Pramanik A. (2021). Workability and flexural properties of fibre-reinforced geopolymer using different mono and hybrid fibres, materials. Materials.

[33] Panda B., Unluer C., Tan M.J. (2019). Extrusion and rheology characterization of geopolymer nanocomposites used in 3D printing. Compos. Part B-Eng..

[34] Bong S.H., Nematollahi B., Xia M., Ghaffar S.H., Pan J.L., Dai J.G. (2022). Properties of additively manufactured geopolymer incorporating mineral wollastonite microfibers. Constr. Build. Mater..

[35] Payakaniti P., Chuewangkam N., Yensano R., Pinitsoontorn S., Chindaprasirt P. (2020). Changes in compressive strength, microstructure and magnetic properties of a high-calcium fly ash geopolymer subjected to high temperatures. Constr. Build. Mater..

[36] Zhang P., Kang L.Y., Zheng Y.X., Zhang T.H., Zhang B. (2022). Influence of SiO_2_/Na_2_O molar ratio on mechanical properties and durability of metakaolin-fly ash blend alkali-activated sustainable mortar incorporating manufactured sand. J. Mater. Res. Technol..

[37] Kan L.L., Wang F. (2022). Mechanical properties of high ductile alkali-activated fiber reinforced composites incorporating red mud under different curing conditions. Ceram. Int..

[38] Kan L.L., Wang F., Zhang Z., Kabala W., Zhao Y.J. (2021). Mechanical properties of high ductile alkali-activated fiber reinforced composites with different curing ages. Constr. Build. Mater..

[39] Gholampour A., Ho V.D., Ozbakkaloglu T. (2019). Ambient-cured geopolymer mortars prepared with waste-based sands: Mechanical and durability-related properties and microstructure. Compos. Part B-Eng..

[40] Yeddula B., Karthiyaini S. (2020). Experimental investigations and GEP modelling of compressive strength of ferrosialate based geopolymer mortars. Constr. Build. Mater..

[41] Sun S. (1984). Adjustment and calculation of sodium silicate modulus. Technol. Build. Well.

[42] Zhang P., Gao Z., Wang J., Guo J.J., Wang T.Y. (2022). Influencing factors analysis and optimized prediction model for rheology and flowability of Nano-SiO_2_ and PVA fiber reinforced alkali-activated composites. J. Clean. Prod..

[43] (2016). Stantard for Test Method of Performance of Ordinary Fresh Concrete.

[44] Li F.Y., Shi T.S., Wang D.H. (2000). Investigation on the thixotropy of concrete. Concrete.

[45] (2009). Standard for Test Method of Basic Properties of Construction Mortar.

[46] Shah S., Chen B., Oderji S.Y., Haque M.A., Ahmad M.R. (2020). Comparative study on the effect of fiber type and content on the performance of one-part alkali-activated mortar. Constr. Build. Mater..

[47] Gao Z., Zhang P., Guo J., Wang K. (2021). Bonding behavior of concrete matrix and alkali-activated mortar incorporating Nano-SiO_2_ and polyvinyl alcohol fiber: Theoretical analysis and prediction model. Ceram. Int..

[48] Zhang H., Sarker P.K., Wang Q.Y., He B., Jiang Z.W. (2021). Strength and toughness of ambient-cured geopolymer concrete containing virgin and recycled fibres in mono and hybrid combinations. Constr. Build. Mater..

[49] Kadhim S., Cevik A., Nis A., Bakbak D., Aljanabi M. (2021). Mechanical behavior of fiber reinforced slag-based geopolymer mortars incorporating artificial lightweight aggregate exposed to elevated temperatures. Constr. Build. Mater..

[50] Hu C.F., Li L., Li Z.L. (2022). Effect of fiber factor on the workability and mechanical properties of polyethylene fiber-reinforced high toughness geopolymers. Ceram. Int..

[51] Zhang P., Wang K., Wang J., Guo J., Ling Y. (2021). Macroscopic and microscopic analyses on mechanical performance of metakaolin/fly ash based geopolymer mortar. J. Clean. Prod..

[52] Seifan M., Mendoza S., Berenjian A. (2020). Mechanical properties and durability performance of fly ash based mortar containing nano- and micro-silica additives. Constr. Build. Mater..

[53] Memon F.A., Nuruddin M.F., Demie S., Shafiq N. (2012). Effect of superplasticizer and extra water on workability and compressive strength of self-compacting geopolymer concrete. Res. J. Appl. Sci. Eng. Technol..

[54] El-Hassan H., Ismail N. (2018). Effect of process parameters on the performance of fly ash/GGBS blended geopolymer composites. J. Sustain. Cem.-Based Mater..

[55] Luukkonen T., Abdollahnejad Z., Ohenoja K., Kinnunen P., Illikainen M. (2019). Suitability of commercial superplasticizers for one-part alkali-activated blast-furnace slag mortar. J. Sustain. Cem.-Based Mater..

[56] Bong S.H., Nematollahi B., Nazari A., Xia M., Sanjayan J. (2019). Efficiency of different superplasticizers and retarders on properties of ‘one-part’ fly ash-slag blended geopolymers with different activators. Materials.

[57] Han Q.Y., Zhang P., Wu J.J., Jing Y.T., Zhang D., Zhang T.H. (2022). Comprehensive review of the properties of fly ash-based geopolymer with additive of Nano-SiO_2_. Nanotechnol. Rev..

[58] Zerfu K., Ekaputri J.J. (2021). Bond strength in PVA fibre reinforced fly ash-based geopolymer concrete. Mag. Civ. Eng..

[59] Assaedi H. (2021). The role of Nano-CaCO_3_ in the mechanical performance of polyvinyl alcohol fibre-reinforced geopolymer composites. Compos. Interface.

[60] Li F.P., Chen D.F., Yang Z.M., Lu Y.Y., Zhang H.J., Li S. (2022). Effect of mixed fibers on fly ash-based geopolymer resistance against carbonation. Constr. Build. Mater..

[61] Zhang P., Han X., Hu S.W., Wang J., Wang T.Y. (2022). High-temperature behavior of polyvinyl alcohol fiber-reinforced metakaolin/fly ash-based geopolymer mortar. Compos. Part B-Eng..

[62] Zidi Z., Ltifi M., Zafar I. (2020). Synthesis and attributes of Nano-SiO_2_ local metakaolin based-geopolymer. J. Build. Eng..

